# Work-related stress and associated factors among garment workers in Bole Lemi Industrial Park of Addis Ababa, Ethiopia: a multi-center institution-based cross-sectional study

**DOI:** 10.1186/s12888-022-04460-7

**Published:** 2022-12-19

**Authors:** Tsegaye Adane Birhan, Muche Ambissa, Tadesse Guadu Delele, Henok Dagne

**Affiliations:** Department of Environmental and Occupational Health and Safety, Institute of Public Health, College of Medicine and Health Sciences, University of Gondar, Gondar, Ethiopia

**Keywords:** Addis Ababa, Ethiopia, Garment workers, Work-related stress

## Abstract

**Background:**

Work-related stress (WRS) is a growing problem around the world that affects the health and well-being of employees. However, evidence of work-related stress in Ethiopia, particularly among garment industry employees, is scarce. This study aimed to assess the prevalence and associated factors of work-related stress among garment workers in the Bole Lemi industrial park in Addis Ababa, Ethiopia.

**Methods:**

A multi-center institutional-based cross-sectional study was conducted from February 01 to March 31, 2021. A total of 605 study participants were selected using a stratified random sampling technique. A pre-tested, interviewer-administered standardized questionnaire was used to collect data. The logistic regression model was employed to identify determinant factors for work-related stress, and statistical significance was declared at a *p*-value < 0.05.

**Results:**

The magnitude of work-related stress in garment workers was 44.1% (95% CI: 40.0, 48.0). Being within the age group of 25 - 34 years (AOR = 0.57, 95% CI: 0.36, 0.90) and receiving induction training (AOR = 0.35, CI: 0.20, 0.62) were protective factors with stress. Whereas lack of family support (AOR = 2.72, CI: 1.60, 4.64), lack of job satisfaction (AOR = 4.26, CI: 2.77, 6.57), and khat chewing (AOR = 3.14, CI: 1.35, 7.32) were positively associated with work-related stress.

**Conclusion:**

Work-related stress is prevalent among garment workers in the study setting. Being between the ages of 25-34 years and receiving induction training were protective factors against stress, while a lack of family support, a lack of job satisfaction, and khat chewing practices were positively associated with work-related stress. Therefore, improving family support and job satisfaction, providing induction training during employment, and providing psychological support for substance users to bring about changes are mandatory in Bole Lemi Industrial Park, Ethiopia.

## Introduction

Work-related stress (WRS) is a pattern of physiological, emotional, cognitive, and behavioral reactions to some extremely demanding aspects of work content, work organization, and work environment [1]. Over the past two decades, the experience of stress at work has had undesirable effects, both on the health and safety of workers and the health and effectiveness of their organizations [2].

People feel stressed when they believe there is a disparity between the expectations placed on them and their ability to meet those needs [3]. Common factors in WRS include lack of control over work, unsuitable demands being made of workers, lack of support from colleagues and management [3, 4], conflict with co-workers or bosses, constant change, and threats to job security, such as potential redundancy [5, 6].

In recent decades, globalization, global competitive processes, and technological progress have transformed the world of work, introducing new forms of work organization, working relations, and employment patterns and contributing to the increment of WRS and its related illnesses [2]. Stress at work has an impact at the individual level, with health effects ranging from coronary heart disease to depression, as well as at the organizational level, reflected in increased absenteeism, reduced productivity, and staff turnover [4].

In many least-developed and developing countries, the garment sector has been a launching pad for progress [7]. There are presently 60-70 million garment employees worldwide, with 75% of them being women. The great majority of these employees labor in informal jobs that are not recognized, regulated, or otherwise safeguarded by the law [8].

The garment industry is among the priority sectors identified under the second growth and transformation plan of Ethiopia [9, 10]. The creation of decent job opportunities, increased exports, ensuring social and environmental sustainability, the creation of industrial linkages, and the transfer of skills and technology are the key prioritized objectives of industrial parks in Ethiopia [9]. Garment industry employers are susceptible to various stress conditions, including excessive overtime, low wages, poor access to social security provisions, verbal and sexual harassment, and the occurrence of forced labor [7].

Furthermore, the uncertain economic crisis places increasing pressure on both employers and workers to remain competitive. Numerous of these transitions present chances for improvement. However, when mismanaged, these shifts increase the risk of psychological disorders and have detrimental effects on health and safety [1, 3]. Challenges and stress in the workplace are usually confusing. While a challenge at work may benefit an individual, work stress is a health concern that might have an impact on occupational safety and health.

Findings suggest that stressful working environments and situations may have an impact on employees’ physical and mental well-being [2]. In Ethiopia, studies on work-related stress are scarce and have almost focused on health sectors. Some studies done among healthcare workers revealed that the prevalence of workplace stress ranges from 46.9 to 68.2% [11–13]. According to a few studies conducted in Ethiopia to measure the prevalence of work-related stress (WRS) and its determinants among employees of the manufacturing sector showed work-related stress ranged from 40.4 to 45.2% [14–16].

Despite the appearance of benefits, employers in the garment sector are frequently faced with excessive overtime, low pay, limited access to social security benefits, verbal and sexual assault, the frequency of bonded labor, and terrible workplace conditions [10, 17]. Studies on work-related stress are still few in the manufacturing industry of the country, despite the existence of a variety of literature on the subject. Additionally, there is little published research on the magnitude of work-related stress among garment workers in Ethiopia or its contributing causes. Considering Bole Lemi Industrial Park is the pioneer government-built industrial park focused on the garment sector, this study aimed to determine the prevalence and identify factors associated with work-related stress among garment workers in Bole Lemi Industrial Park in Addis Ababa, Ethiopia.

## Materials and methods

### Study area and setting

The study was conducted in Bole Lemi Industrial Park’s Addis Ababa, Ethiopia. Bole Lemi is Ethiopia’s first industrial park developed by the Industrial Park Development Corporation. All of the pre-erected factories in Bole Lemi Phase 1 (156 ha), which began operations in 2014, have already been rented out to more than 11 different businesses, employing a total of more than 18,549 workers [18]. This study was done in six garment manufacturing corporations that are under the Bole Lemi industrial park, namely Arvind Lifestyle and Apparel Manufacturing PLC, Ashton Apparel Manufacturing PLC, C & H Garment PLC, Jay Mills Garment PLC, LYU Shoutao Factory PLC, and New Wide Garment Ethiopia Branch Company.

### Study design and period

The multi-center institution-based cross-sectional study design was employed from February 01 to March 31, 2021, in Bole Lemi Industrial Park, Addis Ababa, Ethiopia.

### Source and study population

The source population of the study was all garment workers in Bole Lami industrial park, and the study population was all garment workers who were at work during the period of data collection in Bole Lemi industrial park.

### Inclusion and exclusion criteria

Workers who were currently engaged in the garment industry, who have a minimum of 6 months of work experience, and who were willing to participate in the study were included. Workers who were seriously ill or/and unable to communicate and those who were absent during the data collection period were also excluded.

### Sample size determination and sampling procedure

The sample size was determined using the single population proportion formula, and the 50% proportion was taken as there was no similar study done in the garment industry in the study setting. By considering a 95% confidence level, 5% marginal error, 5% non-response rate, and 1.5 design effect, the total sample size was 605 workers.

A simple random sampling technique was used to select the six garment manufacturing corporations among the eleven. Then, the garment workers were stratified by the work departments, namely Management and administration unit (Ni_1_ = 1794), Finishing unit (Ni_2_ = 1862), Quality control unit (Ni_3_ = 2086), Sewing unit (Ni_4_ = 3746), Cutting unit (Ni_5_ = 2198), and Design and marking unit (Ni_6_ = 1884). Then, the samples were proportionally allocated to each stratum. Then, the required sample sizes were selected by applying a simple random sampling technique in each stratum.

### Data collection tool and procedure

Data were collected using a standard questionnaire adopted from an occupational stress scale developed by the Health and Safety Executive (HSE) of the United Kingdom [19]. Socio-demographic and organizational characteristics, job satisfaction, and behavioral factor-related information were collected. The tool is available in English and translated to the local language, Amharic, and then back to English to check its consistency. Then, the interview was made for selected employees.

### Study variables

#### Dependent variable

Work-related stress (Yes, No).

#### Independent variables

These include socio-demographic characteristics (sex, age, marital status, religion, educational status, monthly income, family support, and work experience), behavioral factors (alcohol consumption, smoking cigarette, and chat chewing), and organizational factors (job demand, job support, job control, role ambiguity, change in organizations, relationships at work, work department, job satisfaction, work shift, and excessive daytime sleepiness).

### Operational definitions

#### Work-related stress (WRS)

It is the reaction that individuals may have when there is an imbalance between the demands of the job and their resources and capacity to fulfill those needs [20]. The HSE-developed occupational stress scale, which has 35 questions, was used for the assessment of WRS. A scoring scale from one to five points is as follows: 1 (never), 2 (rarely), 3 (sometimes), 4 (often), and 5 (always). Two categories were created out of the entire work-related scale. According to the mean score of the total work-related stress, participants who scored above the mean were diagnosed as having work-related stress, and those who scored below the mean were classified as having no work-related stress [21].

#### Work schedule

It includes **s**hift work, night shifts, inflexible work schedules, unpredictable hours, and long or unsociable hours [22].

#### Job satisfaction

A state of pleasurable emotional feeling reported by the worker as the result of one’s job. Job satisfaction was measured by a job satisfaction scale containing 10 Likert scale questions. If an employee’s overall score on the general job satisfaction scale was 32 or higher, they were deemed to be in a satisfactory position [23].

#### Excessive daytime sleepiness (EDS)

It was assessed using the Epworth daytime sleepiness scale [24, 25]. Using a 4-point scale with values ranging from 0 (would never doze) to 3 (high possibility of dozing), respondents rate their propensity to nod off in each of the eight scenarios listed by the scale. The overall score might be between 0 and 24. A person was classified as normal (unlikely to experience daytime sleepiness) if they scored 0–7, borderline (experience an average level of daytime sleepiness) if they scored 8–10, and abnormal (experience excessive daytime sleepiness) if they scored greater than 10.

#### Overtime work

An employee was considered as working overtime when she/he had worked on average greater than or equal to 2 hours more than the standard working hours (48 hours) per week within the past 3 months [26].

#### Ever smoker

Defined as an employee who reported smoking any tobacco products, such as cigarettes, shisha, cigars, or pipes, or hand-rolled cigarettes once in his/her life.

#### Current smoker

Defined as an employee who reported smoking any tobacco products, such as cigarettes, shisha, cigars, or pipes, or hand-rolled cigarettes daily or non-daily irrespective of the quantity within the last 1 month.

#### Alcohol drinker

It is the consumption of any kind of alcohol by a worker at least two times per week [27].

#### Ever Khat chewer

Defined as an employee with the practice of chewing Khat ever in his/her life history.

#### Current Khat chewer

Define as an employee with the practice of chewing Khat in the last 1 month.

### Data quality control

The pretest was done in the Bahir Dar Textile Factory located in Bahir Dar, Ethiopia. The training was also given to the data collectors and supervisors about the objective of the study, data collection, ethical aspects, and methods of communication before they were deployed to do actual work. During data collection, close supervision was made by observing how the data collectors administer the questions to the respondents, and the collected data were checked for completeness, accuracy, and clarity.

### Data management and analysis

The collected data were entered and cleaned using EPI INFO version 7 and exported into STATA 14 for analysis. The descriptive analysis, such as frequency distribution, proportions, percentages, and measures of central tendency were calculated. For the outcome, a variable reliability test was done, and the Cronbach alpha result was 0.789. Multicollinearity between the candidate variables was checked by using the Variance Inflation Factor (VIF) at a cut-off point of 10. Bivariable and multivariable analysis was done to identify factors associated with the outcome variable; statistical significance was declared at *p*-value < 0.05, and odds ratio with 95% confidence levels. For associated factors, model fitness was checked, and the Hosmer-Lemeshow test result was 0.737.

## Results

### Sociodemographic characteristics of garment workers

In the study, 587 (97.02%) garment workers completed the interview. The age of respondents ranges from 18 to 41 years, with a mean age of 25.53 (SD ± 3.62) years. More than half, 329 (56.0%) were in the age group of 25–34 years, 449 (76.5%) were women, 342 (58.3%) were single, and 228 (38.8%) were married. Half of them 290 (49.4%) were diploma/certificate holders. Regarding their work experience, almost all 583 (99.3%) of them served ≤5 years, while 157 (26.7) participants were working in the sewing section (Table 1).

**Table 1:** Socio-demographic Characteristics of garment workers of Bole Lemi industrial parks in Addis Ababa City Administration, 2020 (*n* = 587)

Variables	Category	Frequency (n)	Percent (%)
Sex	Male	138	23.5
	Female	449	76.5
Age	18-24	249	42.4
	25-34	329	56.0
	≥35	9	1.5
Religion	Orthodox	345	58.8
	Muslims	114	19.4
	Protestant	114	19.4
	Others	14	2.4
Marital status	Single	342	58.3
	Married	228	38.8
	Divorced/widowed/separated	17	2.9
Educational status	Primary school	39	6.6
	Secondary school	155	26.4
	Diploma/ Certificate holders	290	49.4
	Degree and above	103	17.5
Monthly salary	≤1400	165	28.1
	1401-1900	135	23.0
	1901-2800	155	26.4
	> 2801	132	22.5
Work experience	≤5 years	583	99.3
	> 5 years	4	0.7
Family size	≤3	323	79.8
	4-6	68	16.8
	≥7	14	3.3
Work section	Design and marking	92	15.7
	Cutting	82	14.0
	Sewing	157	26.7
	Quality control	77	13.1
	Finishing	96	16.4
	Management and admin.	83	14.1

### Behavioral and organizational characteristics

Of the 587 study subjects, 132 (22.49%) of them consumed alcohol at least two times per week and 37 (6.3%) had practiced khat chewing at least once in their lifetime. Moreover, 16 (2.72%) of the respondents were smokers at least once in their lifetime.

From the study participants, almost all 578 (98.5%) respondents were permanent employees. The majority of the respondents 484 (82.5%) of respondents have attended induction training during their employment and 334 (56.9%) of them receive occupational safety and health (OSH) training. Regarding working hours, 520 (88.6%) of the participants worked ≤48 h/week. Two hundred fifty-nine (44.12%) and 261 (64.3%) of the participants experienced a work-related injury in the last 1 year. Accordingly, 394 (67.1%) of the respondents were satisfied with their job (Table 2).

**Table 2:** Behavioral and organizational factors of study respondents in Bole Lemi industrial parks Addis Ababa City Administration, Ethiopia, 2020 (*n* = 587)

Variables	Categories	Frequency (n)	Percent (%)
Alcohol users	Yes	132	22.49
	No	455	77.5
Khat users	Yes	37	6.3
	No	550	93.7
Smokers	Yes	18	3.07
	No	569	96.9
Excessive daytime sleepiness	Normal	513	87.4
	Borderline	37	6.3
	Abnormal	37	6.3
Type of employment	Temporary	9	1.5
	Permanent	578	98.5
Receiving induction training	Yes	484	82.5
	No	103	17.5
Having occupational safety and health training	Yes	334	56.9
	No	253	43.1
Working hours per a week	≤48 hours	520	88.6
	> 48 hours	67	11.4
Tea break (working break)	< 15 minutes	15	2.6
	> 15 minutes	30	5.1
	None	542	92.3
Work-related injury	Yes	259	44.12
	No	328	55.87
Job satisfaction	Satisfied	394	67.1
	Not satisfied	193	32.9

### The magnitude of work-related stress

The magnitude of work-related stress among garment workers of Bole Lemi Industrial Park was 44.1% (95% CI: 40.0, 48.0) in the past 6 months.

### Factors associated with work-related stress among garment workers

Based on the multivariable analysis, being between the ages of 25-34 and receiving induction training were protective factors of WRS, whereas lack of family support, lack of job satisfaction, and khat chewing were positively associated with work-related stress.

Workers within the age group of 25-34 years were 43% less likely to develop work-related stress compared to the age group 18 -24 years (AOR = 0.57, 95% CI: 0.36, 0.90). Workers who had no family support were about 3 times more likely to develop work-related stress compared to those who had family support (AOR = 2.72, 95% CI: 1.60, 4.64). Workers who received induction training during their employment were 65% less likely to develop work-related stress compared to those who had no training (AOR = 0.35, 95% CI: 0.20, 0.62).

Furthermore, the chance of developing work-related stress among current khat chewers was about three times more likely to develop work-related stress compared to those who do not use khat (AOR = 3.14, 95% CI: 1.35, 7.32). Respondents who were dissatisfied with their work were about 4 times more likely to develop work-related stress compared to respondents who were satisfied with their job (AOR = 4.26, 95% CI: 2.76, 6.57) (Table 3).

**Table 3:** Factors associated with work-related stress among garment workers in Bole Lemi industrial parks Addis Ababa City Administration, Ethiopia, 2020 (*n* = 587)

Variable	Work-related stress		COR (95% CI)	AOR (95% Cl)
	Yes	No		
**Age group**				
18-24	139 (23.68%)	110 (18.74%)	1	1
25-34	117 (19.93%)	212 (36.12%)	0.55 (0.44 - 0.69)	0.57 (0.36 - 0.90)*
≥ 35	3 (0.51%)	6 (1.02%)	0.50 (0.13 – 2.00)	0.43 (0.09 – 1.96)
**Marital status**				
Single	170 (28.96%)	172 (29.30%)	1	1
Married	83 (14.14%)	145 (24.70%)	0.57 (0.44 – 0.75)	0.98 (0.63-1.53)
Divorced/widowed/separated	6 (1.02%)	11 (1.88%)	0.55 (0.20 – 1.48)	1.81 (0.65-5.01)
**Educational status**				
Primary	27 (4.60%)	12 (2.04%)	2.25 (1.14 – 4.44)	1.91 (0.60-6.06)
Secondary	78 (13.29%)	77 (13.12%)	1.01 (0.74 – 1.31)	1.25 (0.54-2.89)
Diploma	113 (19.25%)	177 (30.15%)	0.64 (0.50 – 0.81)	1.28 (0.63-2.61)
Degree and above	41 (6.99%)	62 (10.56%)	1	1
**Work department**				
Design & marking	35 (5.96%)	57 (9.71%)	0.74 (0.40 – 1.39)	1.91 (0.72-3.92)
Cutting	38 (6.47%)	44 (7.49%)	0.87 (0.56 – 1.33)	1.52 (0.64-3.60)
Sewing	78 (13.29%)	79 (13.46%)	0.99 (0.72 – 1.35)	1.89 (0.89-4.02)
Quality control	32 (5.45%)	45 (7.67%)	0.71 (0.45 – 1.12)	1.50 (0.66-3.43)
Finishing	50 (8.52%)	46 (7.84%)	1.09 (0.73 – 1.62)	1.91 (0.83-4.43)
Management and administration	26 (4.43%)	57 (9.71%)	1	1
**Family support**				
No	80 (13.63%)	39 (6.64%)	3.31 (2.16 – 5.07)	2.72 (1.60 – 4.64)***
Yes	179 (30.50%)	289 (49.23%)	1	1
**Monthly salary**				
≤ 1400 ETB	84 (14.31%)	74 (12.61%)	1.14 (0.83 – 1.55)	0.99 (0.41-2.37)
1401-1900 ETB	65 (11.07%)	73 (12.44%)	0.89 (0.64 -1.24)	1.23 (0.59-2.59)
1901-2800 ETB	61 (10.39%)	97 (16.52%)	0.63 (0.46 – 0.87)	0.88 (0.45-1.73)
> 2800 ETB	49 (8.35%)	84 (14.31%)	1	1
**Induction training**				
Yes	184 (31.34%)	300 (51.11%)	0.23 (0.14 - 0.37)	0.35 (0.20 - 0.62)***
No	75 (12.78%)	28 (4.77%)	1	1
**Working hours per week**				
≤ 48 hours	297 (50.60%)	223 (37.99%)	1	1
> 48 hours	31 (5.28%)	36 (6.13%)	1.55 (0.93 - 2.58)	1.03 (0.55-1.94)
**Overtime work**				
Yes	219 (37.31%)	303 (51.62%)	0.45 (0.27 - 0.77)	0.89 (0.52 – 1.51)
No	40 (6.81%)	25 (4.26%)	1	1
**Work break**				
≥ 15 minutes	14 (2.39%)	16 (2.72%)	1	1
< 15 minutes	11 (1.87%)	4 (0.68%)	2.21 (1.30 - 3.71)	1.20 (0.31 – 4.63)
None	303 (51.62%)	239 (40.72%)	0.90 (0.43-1.88)	1.01 (0.77 – 1.33)
**Chat chewing**				
No	236 (40.21%)	317 (54.00%)	1	1
Yes	23 (3.92%)	11 (1.87%)	2.81 (1.34 – 5.87)	3.14 (1.35 – 7.32)**
**Job Satisfaction**				
Not satisfied	137 (23.34%)	56 (9.54%)	5.45 (3.74 – 7.95)	4.27 (2.77 - 6.58)***
Satisfied	122 (20.78%)	272 (46.34%)	1	1
**Excessive daytime sleepiness**				
Normal	217 (36.97%)	296 (50.43%)	1	1
Borderline	19 (3.24%)	18 (3.06%)	1.44 (0.74 - 2.81)	1.21 (0.96 – 1.52)
Abnormal	23 (3.92%)	14 (2.38%)	2.24 (1.13 - 4.46)	1.26 (0.99 – 1.60)

*Note*: *ETB* Ethiopian Birr (1ETB = 0.025$, on March 05, 2021)

***p < 0.001, **p < 0.01, and *p < 0.05

## Discussion

Based on the finding, 44.1% of respondents claimed to experience work-related stress. The prevalence of this study was higher than compared to the study conducted in Dukem Ethiopia 40.4% [14], in the Democratic Republic of Congo 28% [28], in India 26% [5], and in Bangladesh 22.9% [29]. The possible reason for this might be brought on by the disparity in socioeconomic status, the level of safety precautions, and better accessibility of health and safety training in different nations [30]. Furthermore, it could also be attributed to the difference in measurement tools used to assess work-related stress, for instance, in Congo Karasek & Siegrist’s scale [28], in Bangladesh ERIs questionnaire [29], in India a tool developed by the National Institute of Mental Health and Neuro Sciences (NIMHANS) [5], and in Dukem, Ethiopia the Work Place Stress Scale (WPSS), the Job Content Questionnaire (JCQ), and Generic Job Stress Questionnaire developed by the National Institute for Occupational Safety and Health (NIOSH) have been used [14]. Work-related stress in an organization has a profound impact on the performance of employees and their productivity, and it affects their health drastically [31].

Being between the age of 25-34 and receiving induction training were significant protective factors of work-related stress, while lack of family support, lack of job satisfaction, and khat chewing were the factors positively associated with work-related stress.

The finding showed that workers between the ages of 25 - 34 years were less likely to develop work-related stress compared to the younger ones. The finding was consistent with those of studies conducted in the Democratic Republic of Congo [28, 32], Bangladesh [29], Japan [6], India [33], and Thailand [34]. The ability to control organizational and work-related stress was found to get better with age, thus that might be the cause. It could also be that they are more versatile in the workplace than their counterparts, which enables them to change and adapt to the organizational culture or quickly pick up new skills and processes, and develop improved job skills and work experience [5].

According to this study, workers who lacked family support were about three times more likely to develop work-related stress compared to those who had family support. This finding was in line with a study conducted in Bangladesh [35, 36] and Thailand [34]. This might be due to the lack of family support, which may discourage workers from discussing any incongruent demands between work and home, diminishing job satisfaction and worker commitment, and aggravating stress [37, 38]. This suggests that families can help working people fulfill their job responsibilities, which may in turn serve to promote their career objectives, improve family contentment, and minimize stress.

In this study, workers who received induction training were about 65% less likely to develop work-related stress compared to those who had no induction training. The finding was similar to other studies [29, 39]. Employees’ speedy adaptation to the new working environment is facilitated by induction training. Additionally, the organizational setting facilitates and speeds up the social and psychological adjustment of new hires. It reduces workplace stress and makes workers more tolerant of stress, which is beneficial [39].

Participants who were dissatisfied with their work were about four times more likely to develop WRS than respondents who were satisfied with their job. The finding was consistent with the studies conducted in Mekelle City [12] and India [33]. The reason might be that dissatisfied workers have poor interaction and communication with their colleagues and supervisors/managers and less coping ability [12].

The chance of developing work-related stress among current khat chewers was three times more likely to develop work-related stress compared to those who do not chew khat. This finding was in line with a study conducted in Jimma [11]. This might be because khat chewing may lead to loss of working hours or absenteeism from work, impaired or altered memory, and loss of concentration at work, which may result in decreased productivity and loss of job [40]. As a result, as people chew khat more frequently, they may feel ashamed and blame themselves, which over time results in dissatisfaction and stress.

### Limitations of the study

Due to its cross-sectional study design, this study could not show a temporal relationship. Since the study was self-reported, the results might be subjected to either over/underreporting due to recall bias.

## Conclusion

This study showed that work-related stress was prevalent among garment workers. Being between the age of 25 - 34 years and receiving induction training were protective factors of work-related stress, while lack of family support, lack of job satisfaction, and being a current khat chewer were the factors positively associated with work-related stress. Therefore, it needs to set up intervention programs focusing on the prevention and control of stress through tailored interventions targeted at improving the family and social support of employees, job satisfaction, the provision of induction training during employment, and providing psychological support for substance users, especially khat chewers, to bring about changes.

## Data Availability

The dataset of this research is available upon reasonable request from the corresponding author.
